# The effect of virtual reality technology use on satisfaction with training matches for university basketball players: a mediated chain effect of self-efficacy and sport engagement

**DOI:** 10.3389/fpsyg.2026.1787384

**Published:** 2026-03-26

**Authors:** Jigang Li, Feng Zhao, Hefei Ma

**Affiliations:** 1 School of Physical Education, Northeast Normal University, Changchun, China; 2 School of Physical Education and Health, Yili Normal University, Yili, China

**Keywords:** chain mediating effects, satisfaction with training matches for UBPs, self-efficacy, sport engagement, university basketball players, virtual reality (VR) technology

## Abstract

**Background:**

Against the backdrop of deep integration between scientific and digital approaches in sports training, players’ mental wellbeing has garnered increasing attention. Virtual reality (VR) technology, with its immersive, interactive and conceptual advantages, offers an innovative supplementary tool for training matches among university basketball players (UBPs).

**Objectives:**

This study aims to investigate the chained mediating effects of self-efficacy and sport engagement on VR technology use and satisfaction with training matches for UBPs.

**Methodology:**

A total of 482 valid responses were collected using the VR technology use scales, satisfaction with training matches for UBPs, self-efficacy scales and sport engagement scales.

**Results:**

The analysis of mediating effects in this study indicates that self-efficacy and sport engagement exert a significant mediating influence on the pathway linking VR technology use to satisfaction with training matches for UBPs. The effect size for the total indirect effect is 0.225 (95% CI [0.145–0.286]). This mediating effect comprises three distinct pathways. Path 1: VR technology use → Self-efficacy → satisfaction with training matches for UBPs, effect size 0.100, 95% CI [0.065–0.132]. Path 2: VR technology use → sport engagement → satisfaction with training matches for UBPs, effect size 0.104, 95% CI [0.071–0.128]. Path 3: VR technology use → self-efficacy → sport engagement → satisfaction with training matches for UBPs, effect size 0.021, 95% CI [0.005–0.041].

**Conclusion:**

The VR technology use is positively correlated with the satisfaction with training matches for UBPs. This chain of mediating effects may be realised by stimulating self-efficacy and enhancing their sport engagement of UBPs.

## 1 Introduction

Higher education institutions serve as vital breeding grounds for basketball talent. Many outstanding basketball players have emerged from university teams to pursue professional careers (Bond et al., 2019). Participation in basketball training and competition constitutes an essential component of students’ holistic development (Heishman et al., 2021). The sport demands players possess excellent physical conditioning, refined technical skills, and outstanding teamwork abilities. During training, physical fitness undergoes significant enhancement. Simultaneously, through seamless coordination with teammates and intense competition against opponents, their communication skills, teamwork awareness, and mental resilience are honed (Yılmaz et al., 2025). However, if players experience dissatisfaction during training and matches, they may gradually lose enthusiasm and motivation for the sport (Evans et al., 2023; Staufenbiel et al., 2018). This may have hindered their progress in basketball, personal growth, and overall development. Currently, research on the satisfaction with training matches for university basketball players (UBPs) remains relatively scarce. This scarcity hinders universities from adequately considering players’ actual circumstances and expectations when formulating basketball training plans and organising match activities, thereby affecting both player satisfaction and team development. Furthermore, previous studies have predominantly focused on ordinary university students and professional players, whereas university basketball players (UBPs) may exhibit distinct psychological characteristics and training environments (Nicholls et al., 2020; Pankow et al., 2021). Given basketball’s nature as a highly competitive team sport, players’ emotional responses are particularly sensitive to external factors such as opponents, spectators, and referees (Mavili et al., 2025). Consequently, this study specifically targets the cohort of UBPs, aiding in the precise identification of the relationship between virtual reality technology and athlete satisfaction. It thereby provides targeted recommendations for the training and management of university sports teams.

In this era of rapid technological advancement, VR technology—as an emerging field within information science in recent years—has found application across numerous domains, particularly within sports training (Stefan et al., 2022). As a vital component of physical education, university basketball has continually explored innovative training methodologies to enhance players’ skill levels and competitive performance. The advent of VR technology has infused university basketball training with renewed vitality, unlocking unprecedented possibilities (Soltani and Morice, 2023). Traditional university basketball training methods are often constrained by factors such as venue limitations, time restrictions, and the skill level of opponents. Consequently, training content and formats tend to be relatively monotonous, struggling to meet players’ individualised training needs. Players may find training tedious, leading to fatigue and disengagement, which ultimately undermines training effectiveness. In contrast, VR technology offers distinct advantages (Wei et al., 2022). It can precisely record user movements and simulate complex, high-risk sporting scenarios, enabling players to undergo high-intensity training in a risk-free environment (Trpkovici et al., 2025). By creating highly realistic match scenarios that immerse players in authentic game conditions, this immersive experience significantly enhances training engagement and interactivity while better preparing players for competitive atmospheres (Jia et al., 2025). In addition, existing research indicates that players’ training satisfaction is influenced by multiple factors, including training methods, psychological state, and environmental support (Yu et al., 2024). However, systematic exploration of the mechanisms underlying satisfaction with training matches for UBPs remains limited. Compared to professional athletes, UBPs typically face dual pressures from academics and training, with relatively limited training resources and specialized support (Gray et al., 2023; Ishii et al., 2018). Yet, compared to general college students, they exhibit stronger demands for specialized skills and greater motivation for competitive performance. This unique position may result in distinctive structures and influencing factors for their training satisfaction (Bennett, 2017). Concurrently, while virtual reality (VR) technology is increasingly applied in athletic training, most research focuses on skill acquisition and motion analysis, with limited exploration from the perspective of athletes’ psychological experiences and satisfaction. Particularly lacking is research examining how VR influences training perceptions among UBPs through psychological mediating mechanisms. Consequently, this study’s in-depth examination of the relationship between VR and satisfaction with training matches for UBPs holds significant theoretical and practical value. Research into this topic reveals players’ needs and expectations during training matches, identified factors that may be associated with satisfaction, and provides a reference for universities to develop scientifically sound training programmes and organise engaging match events. Simultaneously, it helps boost players’ motivation and initiative, fostering the vigorous development of university basketball and cultivating more outstanding talent for the sport.

## 2 Literature review

Satisfaction with training matches could reflect players’ emotional states throughout their training and match engagements (Nicholls et al., 2009). Therefore, factors related to players’ training and competition performance may be directly or indirectly linked to satisfaction (Health Co, Sport Science SCSU-U et al., 2019). As an innovative tool in the sports technology revolution, VR is reshaping traditional paradigms in university basketball training, exerting multidimensional and profound effects on players’ training and competition satisfaction (Bum et al., 2018). Traditional training models exhibit limitations in scenario simulation, personalised feedback, and psychological load regulation. VR technology, however, can provide highly immersive, repeatable, and safely controllable virtual training environments, offering players novel experiences (Kumar et al., 2021). This technology not only may simulate complex match scenarios for tactical decision-making training and technical movement analysis but also may reshape players’ perceptions and evaluations of training from a psychological perspective (Souza et al., 2024). Specifically, through its immersive and gamified design, VR technology may enhance the enjoyment and appeal of the training process. This directly satisfies players’ intrinsic need for novelty and challenge, thereby directly influencing satisfaction levels (Fleming et al., 2023; Kim and Ko, 2019). More importantly, it may contribute to players’ confidence in their technical and tactical abilities, strengthening their inner conviction. Therefore, players engage in training with a more positive, confident, and focused mindset.

Self-efficacy, as a key concept within the field of sports psychology, refers to an individual’s subjective assessment and confidence in their ability to successfully accomplish a particular task (Kaufmann et al., 2022). In university basketball training, players’ self-efficacy exerts connection on their training motivation, skill development, and match performance. When players possess high self-efficacy, they engage more proactively in training, demonstrate greater willingness to attempt challenging techniques and tactics, and perform with increased composure and confidence during matches (Zmora et al., 2026). Self-efficacy significantly predicts players’ exercise behaviour, with those exhibiting high self-efficacy being more likely to set challenging training objectives (Jing, 2023). Maintaining long-term exercise habits and employing proactive coping strategies when facing difficulties also help actively promote participation in sport. A strong sense of self-efficacy among players is one of the key factors in achieving outstanding performance (Deng, 2023). The higher an athlete’s sense of self-efficacy, the greater their level of effort and the higher their satisfaction with training. This perspective was validated in a football programme, where enhanced self-efficacy improved players’ training outcomes and physical and mental wellbeing, as well as comparatively superior match results (Yang, 2005). VR technology, by providing players with diverse, personalised training experiences, may enable them to accumulate successful experiences through the continuous overcoming of challenges, thereby progressively elevating their sense of self-efficacy.

Sport engagement reflects the degree to which players devote themselves fully to training and competition, encompassing aspects such as concentration, perseverance, and emotional investment (Harrison et al., 2024). Under traditional training models, players may struggle to maintain consistently high levels of engagement due to the monotony and repetitiveness of drills. The immersive training environments created by VR technology, however, could capture players’ attention, stimulate their intrinsic motivation, and encourage more proactive participation in training, may enhancing their level of engagement (Dobre et al., 2022). Through VR technology, UBPs can immerse themselves in highly realistic training and match scenarios, stimulating a deeper passion and enthusiasm for the sport. The simulated intensity of competition within the virtual environment—complete with spectator cheers and the palpable tension of the moment—may allow players to experience the allure of the game more profoundly (Gulec et al., 2019). VR technology also provides players with diverse training content and strategic analysis. Within virtual scenarios, players can repeatedly experiment with tactical combinations and shooting techniques, gaining profound insights into basketball’s principles and strategies (Jiang et al., 2022). This cognitive engagement could enhance players’ basketball literacy and competitive prowess, enabling them to perform with greater ease and confidence in both training and matches.

Self-efficacy and sport engagement exert a closely interlinked chain of mediating effects on how VR technology influences satisfaction with training matches for UBPs. Self-efficacy represents players’ assessment of their capacity to successfully execute specific basketball tasks. Sport engagement manifests as their focus, effort, and dedication during training and matches (Liu et al., 2024). Through virtual reality technology, players can simulate complex match scenarios for training; achieving strong performance in these simulations enhances self-efficacy (Stephen et al., 2022). This heightened self-efficacy subsequently motivates players to adopt a more positive attitude towards training and competition, increasing sport engagement (Soto Garcia et al., 2021; Terranova and Henning, 2011). Greater sport engagement may yield improved training outcomes and competition results, further elevating satisfaction with training matches for UBPs. Thus, self-efficacy and sport engagement mutually reinforce each other, collectively forming the bridge between VR technology and satisfaction with training matches for UBPs.

**Figure 1:**
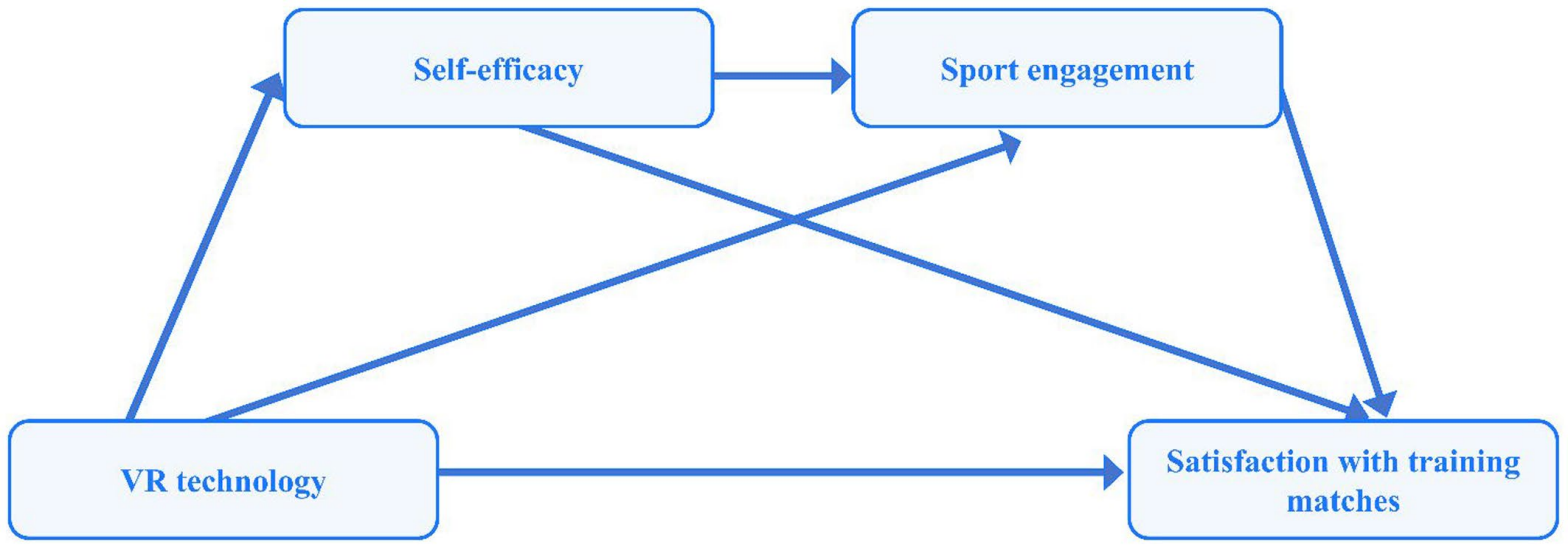
Chain of intermediation hypothesis model.

This study aims to investigate the relationship between VR technology and satisfaction with training matches for UBPs, whilst conducting an in-depth analysis of the chained mediating effects of self-efficacy and sport engagement within this process. As illustrated in Figure 1, the research proposes to construct a chained mediation model, with the following hypotheses:

*H1*: VR technology use exerts a significant positive predictive effect on satisfaction with training matches for UBPs.

*H2*: Self-efficacy plays an independent mediating role between VR technology and satisfaction with training matches for UBPs.

*H3*: Sport engagement plays an independent mediating role between VR technology and satisfaction with training matches for UBPs.

*H4*: Self-efficacy and sport engagement exert a chain-mediated effect between VR technology and satisfaction with training matches for UBPs.

## 3 Method

### 3.1 Research design and participants

This study employed a combination of cluster sampling and purposive sampling to select research subjects. Through systematic screening, university representative teams were identified that had systematically and deliberately incorporated VR training into their weekly training programmes for at least 3 months, applying it to basketball-specific training for at least one complete training cycle. The VR system utilizes a PC-based head-mounted display (HMD) model PICO Neo 3, featuring a 98° field of view (FOV) and 90 Hz refresh rate. It incorporates a built-in 6DoF (six degrees of freedom) positioning system to ensure immersive experiences and precise motion capture. Training content is delivered through customized basketball-specific VR software, comprising two primary modules. The first is tactical scenario decision-making training, simulating common game situations such as screens, drives, and passes, requiring athletes to make real-time judgments and reactions within the virtual environment. The second is shooting mechanics reinforcement training, where the system records the angle, speed, and trajectory of shot releases, providing real-time visual feedback. Ultimately, men’s and women’s basketball representative teams from six universities across eastern (Shanghai, Jiangsu), central (Hubei, Hunan), and western (Sichuan, Shaanxi) China were confirmed as the study population. Prior to implementation, informed consent was obtained from the sports departments of all participating institutions, head coaches, and players themselves. All parties agreed that data would be used solely for academic research, with strict anonymisation and confidentiality protocols applied. A total of 550 questionnaires were distributed. After excluding invalid responses (e.g., abnormally short completion times, discernible patterned responses, or missing key information), 482 valid questionnaires were retrieved, yielding an effective response rate of 87.6%. The demographic and training characteristics of the valid sample were as follows: 368 male players (76.3%) and 114 female players (23.7%); mean age 20.44 ± 3.71 years; mean duration of basketball training 3.25 ± 1.93 years. All players utilised a designated VR basketball training system weekly during the preceding semester for specialised technical/tactical or cognitive training. The average usage frequency was 2.33 ± 0.39 sessions per week, with an average session duration of 40.52 ± 8.46 min. This ensured all participants possessed sufficient VR technology experience to guarantee the validity of measurement outcomes.

This study strictly adheres to the ethical principles outlined in the Declaration of Helsinki and has received formal approval from the Academic Ethics Committee of the School of Psychology at Northeast Normal University. All UBPs invited to participate were fully informed of the study’s objectives, procedures, and potential discomforts. They were explicitly assured of their right to withdraw from the study at any stage without condition, and that this would not adversely affect their training or academic pursuits. All participants in this study were players aged 18 years or older, who personally read and signed written informed consent forms. All collected questionnaire data were processed using anonymous coding. Any personally identifiable information was separated from the research data and kept strictly confidential. The reporting and publication of research findings involved only aggregate data analysis and will never disclose any individual’s private information.

### 3.2 Research tools

#### 3.2.1 VR technology use scales

Each item on this scale has been adapted to suit the training and competition contexts of basketball players, based on existing research by Yang and Zhao (Yang et al., 2019; Zhao, 2022). This scale comprises four dimensional structural variables: presence, perceived usefulness, perceived ease of use, and intention to practise. In this study, a five-point Likert scale was employed to measure respondents’ experiential attitudes towards each dimension. Response options were assigned values of 5, 4, 3, 2, and 1, corresponding to “Strongly Agree,” “Agree,” “Neutral,” “Disagree,” and “Strongly Disagree” respectively. The scale demonstrated a Cronbach’s alpha coefficient of 0.75, *χ*^2^/df = 3.2, CFI = 0.91, TLI = 0.89, RMSEA = 0.07 (Supplementary Table S1).

#### 3.2.2 Satisfaction with training matches scales

The measurement of satisfaction with training matches for UBPs employs Zhang’s scale, which is unidimensional and comprises six items (with Item 5 being reverse-scored) (Zhang, 2022). In this study, a five-point Likert scale was employed for measurement, with higher scores indicating greater satisfaction with the training matches. The Cronbach’s alpha coefficient for this scale was 0.82, *χ*^2^/df = 2.1, CFI = 0.96, TLI = 0.94, RMSEA = 0.05.

#### 3.2.3 Self-efficacy scales

The measurement of self-efficacy among UBPs employs the Self-Efficacy Scale by Schwarzer and Wang et al. Certain items have been adapted to incorporate terminology appropriate to sports science contexts (Schwarzer et al., 1995; Wang et al., 2001). This scale is a unidimensional instrument comprising 10 items, employing a five-point Likert scale. Higher scores indicate greater self-efficacy. We have amended five of the entries: “2. If someone opposes me, I can find the means and ways to get what I want.” has been revised to “2. Even if the coach or teammates have reservations about my choices, I still possess the means to achieve my objectives.” “4. I am confident that I could deal efficiently with unexpected events.” has been revised to “In training matches, as a UBP, I am confident I can effectively handle any sudden occurrences.” “5. Thanks to my resourcefulness, I know how to handle unforeseen situations.” has been revised to “5. In training matches, with my resourcefulness, I am certain I can manage unexpected situations.” “8. When I am confronted with a problem, I can usually find several solutions.” has been revised to “8. When faced with a challenge in training matches, I can typically devise multiple solutions.” “9. If I am in trouble, I can usually think of a solution.” has been revised to “9. When the team faces a scoring drought or consecutive errors, as a UBP I can usually devise ways to help the team overcome the predicament.” During the adaptation process, we systematically contextualised the relevant entries. Compared to general self-efficacy, this sport-specific self-efficacy demonstrates predictive validity for particular behaviours and attitudes. The Cronbach’s alpha coefficient for this scale is 0.86, *χ*^2^/df = 3.8, CFI = 0.93, TLI = 0.91, RMSEA = 0.08, demonstrating sound construct validity and suitability for large-scale measurement.

#### 3.2.4 Sport engagement scales

The Lonsdale sport engagement questionnaire (AEQ) comprises 16 items organised into four dimensions: self-confidence, dedication, vigour, and enthusiasm. Each dimension contains four items (Wan et al., 2025; Lonsdale et al., 2007). In this study, a five-point Likert scale was employed for measurement, with higher scores indicating greater sport engagement. The Cronbach’s alpha coefficient for this scale was 0.79, *χ*^2^/df = 3.5, CFI = 0.92, TLI = 0.90, RMSEA = 0.07.

### 3.3 Data analysis

Data analysis was performed using SPSS 26.0 software. Descriptive statistics (including mean, standard deviation, frequency, and percentage) were employed to characterise the demographic features of the subjects (Zhao et al., 2023). Continuous variables and categorical variables were described separately. Pearson correlation analysis was performed between variables. Coefficients closer to 1 indicate stronger correlation, while those closer to 0 indicate weaker correlation. Excessively high correlations necessitate vigilance for multicollinearity. Multicollinearity was assessed using tolerance (>0.1) and variance inflation factor (VIF < 5) as diagnostic criteria (Chen et al., 2023). Employing the non-parametric percentile bootstrap method via the SPSS PROCESS macro to test for mediating effects (Xiao et al., 2025).

## 4 Results

### 4.1 Common method bias test

Given that this study employed translation scales encompassing different languages for measurement, and all quantitative data were based on participants’ self-reports, there exists a potential risk of common method bias. During data analysis, we conducted confirmatory factor analysis and reliability testing for each scale, utilising Harman’s single-factor method to assess common method bias (Li et al., 2019). Using the factor analysis functionality within SPSS 26.0 software, an unrotated exploratory factor analysis was conducted. The results indicated the presence of seven factors with eigenvalues exceeding 1. The first factor accounted for 31.80% of the variance, which was significantly below the critical threshold of 40%. It may be inferred that the data in this study were not subject to severe common method bias (Liao, 2025).

### 4.2 Descriptive statistics and correlation analysis of the variables

The means, standard deviations, and correlations among variables are presented in Table 1. As shown in Table 1, descriptive statistics reveal that the mean scores for VR technology use (3.72 ± 1.06), self-efficacy (4.76 ± 1.27), sport engagement (3.91 ± 0.94), and satisfaction with training matches for UBPs (2.31 ± 0.43) were distributed as follows. Significant correlations exist between VR technology use, self-efficacy, sport engagement, and satisfaction with training matches for UBPs. Specifically, VR technology usage exhibits a positive correlation with satisfaction with training matches for UBPs (r = 0.42, *p* < 0.05), and also shows positive correlations with self-efficacy (r = 0.41, *p* < 0.01) and sport engagement (r = 0.41, *p* < 0.01). Concurrently, self-efficacy positively correlated with satisfaction with training matches for UBPs (r = 0.44, *p* < 0.01) and with sport engagement (r = 0.33, *p* < 0.01). In addition, sport engagement exhibited a significant positive correlation with satisfaction with training matches for UBPs (r = 0.46, *p* < 0.01).

**Table 1:** Descriptive statistics and correlation analysis results for VR technology use, self-efficacy, sport engagement, and satisfaction with training matches for UBPs.

Variables	Mean	SD	VR technology use	Self-efficacy	Sport engagement	Satisfaction with training matches for UBPs
VR technology use	3.72	1.06	1			
Self-efficacy	4.76	1.27	0.41**	1		
Sport engagement	3.91	0.94	0.41**	0.33**	1	
Satisfaction with training matches for UBPs	2.31	0.43	0.42*	0.44**	0.46**	1

*p < 0.05, **p < 0.01.

### 4.3 Regression analysis of the chain mediation model

Due to significant correlations among variables, multicollinearity issues may arise. Consequently, this study conducted collinearity diagnostics and standardised the predictor variables in each equation (Z-scores). Results indicate that all predictor variables exhibited tolerance ranges of 0.50–0.83 (>0.1) and variance inflation factors (VIF) of 1.27–2.09 (<5). Based on these multicollinearity test results, no severe multicollinearity issues were identified, supporting progression to subsequent mediation and chained mediation effect analyses. The SPSS Process plugin developed by Hayes was employed, selecting Model 6 from the templates. VR technology use was designated as the independent variable, satisfaction with training matches for UBPs as the dependent variable, self-efficacy and sport engagement as mediating variables, and gender and age as control variables. A chained mediation model effect analysis was conducted with 5,000 repeated samples and a 95% confidence interval.

**Figure 2:**
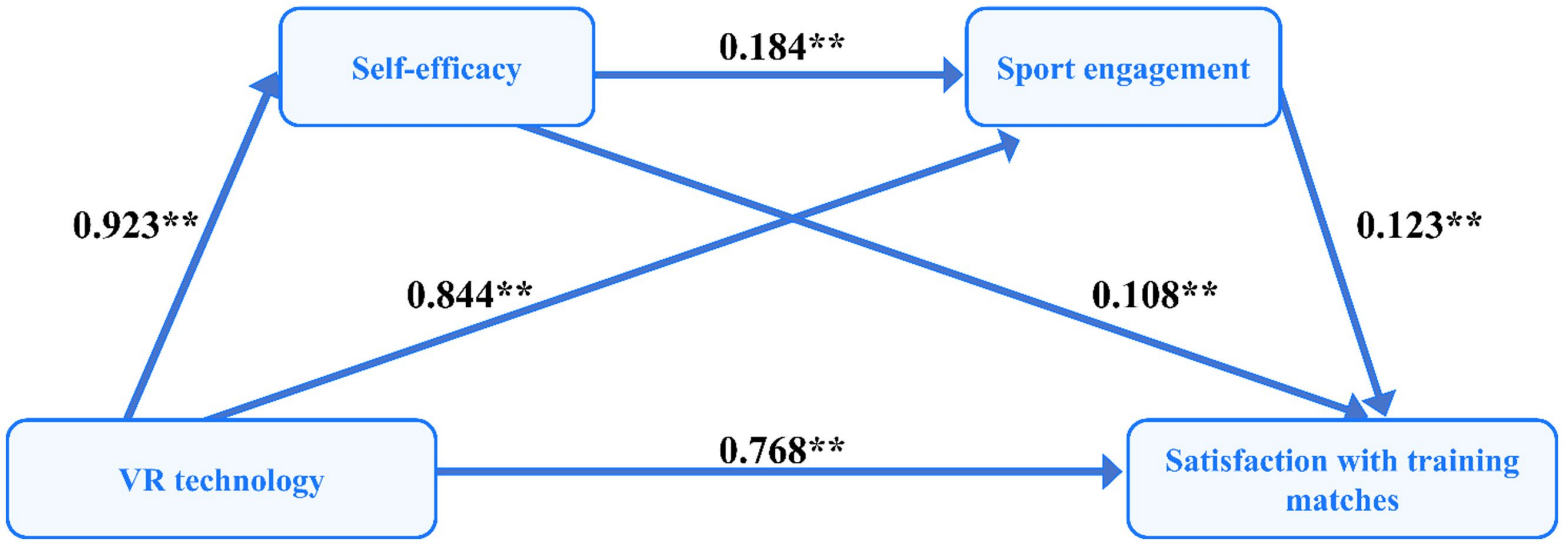
A chained mediation model of self-efficacy and sport engagement in the relationship between VR technology use and satisfaction with training matches for UBPs. The final model revealed standardized regression coefficients between these variables (**p < 0.01).

As shown in Table 2, regression analysis revealed that VR technology use was significantly and positively associated with self-efficacy (*β* = 0.923, *t* = 8.254, *p* < 0.01), sport engagement (*β* = 0.844, *t* = 27.347, *p* < 0.01), and satisfaction with training matches for UBPs (*β* = 0.768, *t* = 31.583, *p* < 0.01). Concurrently, self-efficacy was significantly associated with sport engagement (*β* = 0.184, *t* = 5.506, *p* < 0.01) and satisfaction with training matches for UBPs (*β* = 0.108, *t* = 6.394, *p* < 0.01). Moreover, sport engagement was significantly associated with satisfaction with training matches for UBPs (*β* = 0.123, *t* = 5.473, *p* < 0.01). The overall model demonstrated satisfactory explanatory power, with R^2^ values of 0.132, 0.678, and 0.794, respectively. All *F*-values were statistically significant (*p* < 0.01). As illustrated in Figure 2, this indicates the model provides satisfactory explanatory adequacy.

**Table 2:** Regression analysis of the chain intermediary model.

Variables	Self-efficacy		Sport engagement		Satisfaction with training matches for UBPs	
	*β*	*t*	*β*	*t*	*β*	*t*
VR technology use	0.923	8.254^**^	0.844	27.347^**^	0.768	31.583^**^
Self-efficacy			0.184	5.506^**^	0.108	6.394^**^
Sport engagement					0.123	5.473^**^
Gender	0.147	0.763	−0.014	−0.191	0.048	0.464
Age	−0.175	−0.773	0.088	0.313	0.092	0.559
*R* ^2^	0.132		0.678		0.794	
*F*	126.818^**^		145.324^**^		377.183^**^	

**p < 0.01.

As shown in Table 3, the results of the mediating model effect tests indicate that VR technology use influences the satisfaction with training matches for UBPs, with a direct effect size of 0.768 (95% CI [0.721–0.816]) and a relative effect proportion of 77.3%. Furthermore, it affects satisfaction via the chained mediating effects of self-efficacy and sport engagement, with the mediating effect arising from three indirect pathways: Path 1 (VR technology use → self-efficacy → satisfaction with training matches for UBPs) exhibited a significant indirect effect with an effect size of 0. 100 (95% CI [0.065–0. 132]), accounting for 10.1% of the relative effect. Path 2 (VR technology use → sport engagement → satisfaction with training matches for UBPs) exhibited a significant indirect effect with an effect size of 0. 104 (95% CI [0. 071–0. 128]), accounting for 10.5% of the relative effect. Path 3 (VR technology use → Self-Efficacy → sport engagement → satisfaction with training matches for UBPs) exhibited a significant indirect effect, with an effect size of 0. 021 (95% CI [0. 005–0. 041]) and a relative effect proportion of 2.1%. The total indirect effect size was 0. 225 (95% CI [0. 145–0. 286]), accounting for 22.7% of the relative effect. This indicates that the chained mediating role of self-efficacy and sport engagement was supported within the model examining the influence of VR technology use on satisfaction with training matches for UBPs.

**Table 3:** Mediation effect analysis table.

Effect type	Effect value	Bootstrap 95%CI		Relative effect proportion
		LL	UL	
Total effect	0.993	0.991	0.995	100%
Direct effect	0.768	0.721	0.816	77.3%
Indirect effect 1	0.100	0.065	0.132	10.1%
Indirect effect 2	0.104	0.071	0.128	10.5%
Indirect effect 3	0.021	0.005	0.041	2.1%
Total indirect effect	0.225	0.145	0.286	22.7%

Direct effect pathway: VR technology use → Satisfaction with training matches for UBPs; Indirect effect 1: VR technology use → Self-efficacy → Satisfaction with training matches for UBPs; Indirect effect 2: VR technology use → sport engagement → Satisfaction with training matches for UBPs; Indirect effect 3: VR technology use → Self-efficacy → sport engagement → Satisfaction with training matches for UBPs.

In summary, VR technology use was directly associated with higher the satisfaction with training matches for UBPs, meaning that greater VR technology use correlates with higher satisfaction levels. Furthermore, the application of VR technology use could exert an indirect effect on training match satisfaction through its respective influence on self-efficacy and sport engagement. That is to say, increased VR technology use could positively predict heightened levels of self-efficacy and sport engagement, which in turn contributes to greater satisfaction with training matches for UBPs.

## 5 Discussion

This study employed a chain mediation model to examine the relationship between VR technology use and satisfaction with training matches for UBPs. Particular attention was paid to the mediating roles of self-efficacy and sport engagement. Findings indicate that VR technology use was positively associated with satisfaction with training matches for UBPs, with both self-efficacy and sport engagement playing significant mediating roles in this relationship. All three mediation pathways were statistically significant and supported the chain mediation model.

### 5.1 The direct effect of VR technology use on the satisfaction with training matches for UBPs

VR technology use was positively correlated with satisfaction with training matches for UBPs. Research has found that VR technology, through its immersive, highly realistic, and repeatable virtual environments, not only mechanistically parallels the enhancement of satisfaction through “work engagement” in positive work settings, but also potentially generates more intense and immediate engagement experiences due to its strong attraction of sensory input and attention (Leveau and Camus, 2023). Specifically, when players are immersed in highly realistic virtual basketball arenas, the immersive experience of applying techniques, immediate feedback on situational responses, and the ability to repeatedly practise without physical constraints could enhance the quality of experience and emotional engagement during training (Greenhough et al., 2021). VR technology could create a high-fidelity, low-risk, and highly customisable training environment, enabling players to focus on refining technical details and understanding tactical intentions (Grabowski et al., 2024). This controllability and safety alleviate the anxiety and frustration associated with traditional high-intensity competitive training, enhancing the acceptability and enjoyment of the training process itself. More significantly, VR scenarios can replicate critical plays, pressure moments, or specific opponent tactics that are difficult to recreate in actual matches (Pagé et al., 2019). This may provide players a psychological sense of control and preparedness (Ropponen et al., 2025). For instance, if a defender is within arm’s length of another player, the shooter should not attempt a shot but instead drive to the basket or pass to an open teammate. Unfortunately, even for professional players, making flawless offensive decisions at critical moments remains challenging during highly competitive basketball matches (Tsai et al., 2021). Through VR technology, players can undergo training under well-controlled conditions that simulate real-world competition scenarios (Düking et al., 2018). Repeatable scenarios within VR also afford players boundless practice opportunities and satisfaction with training matches. Arias-Estero et al. organised a tournament to analyse young basketball players’ decision-making capabilities. They noted that while young players require extensive practice to enhance their decision-making skills, opportunities for successful practice within matches remain scarce, resulting in a lack of satisfaction with training matches (Arias-Estero et al., 2018). Raab et al. emphasise that successful performance in sport is determined by the integration of movement type and flawless execution, thereby enhancing players’ satisfaction with training matches (Raab et al., 2005).

### 5.2 The mediating role of self-efficacy in the relationship between VR technology use and satisfaction with training matches for UBPs

Self-efficacy may function as a mediating bridge between VR technology use and satisfaction with training matches for UBPs. Specifically, virtual reality technology, through its immersive, repeatable, and highly controllable characteristics, may help create specific environments for players, thereby systematically constructing and enhancing their self-efficacy (Farrow et al., 2019). VR technology enables the simulation of game-like scenarios within a safe, controlled environment, allowing players to participate in various settings, reflect upon their performance, and receive immediate feedback (Wang and Zhang, 2025). Interaction with virtual environments and heightened immersion foster deeper engagement, thereby enhancing intrinsic motivation (Farrow et al., 2019; McCormick et al., 2019). Moreover, VR could assist players in reducing anxiety, enabling them to adapt to competitive environments without experiencing the tension associated with real matches (Tenforde et al., 2022). Players with high self-efficacy are more inclined to set challenging personal goals and exert greater sustained effort during training and competitions. When confronted with difficulties, they demonstrate greater resilience and creative problem-solving, attributing their performance more to controllable personal abilities and preparation than to external luck or opponent factors (Liu et al., 2024). This positive, internalised attribution approach directly may be associated with a higher valuation of the competition process and greater emotional fulfilment. Research examining the influence of self-efficacy on fencing coaches’ democratic leadership behaviours and players’ training satisfaction indicates that fencers’ self-efficacy and athletic achievement goals mediate the effect of coaching democratic leadership on satisfaction with training matches for UBPs (Dai, 2021). Coaches possessing high self-efficacy may be better able to devise well-structured and effective training programs, potentially enabling players to progress rapidly and achieve satisfaction. Furthermore, such coaches may be better equipped to motivate and support players, helping them proactively tackle challenges. They respect individual differences among players, providing personalised guidance that fosters a sense of being valued and respected (Chao et al., 2023).

### 5.3 The mediating role of sport engagement in the relationship between VR technology use and satisfaction with training matches for UBPs

The findings of this study suggestion that sport engagement plays a mediating role between VR technology use and the satisfaction with training matches for UBPs. Sport engagement constitutes an enduring, positive, cognitive-affective experience within sporting contexts, characterised by self-confidence, dedication, vigour, and enthusiasm (Curran et al., 2015). Sport engagement serves as a relevant indicator for assessing an athlete’s positive psychological state. It could stimulate the development of beneficial qualities, thereby promoting the athlete’s physical and mental wellbeing, and lays a foundation for enhancing their competitive level and performance outcomes (Zhang, 2012). The simulation and resolution of real-match challenges within virtual environments imbue training with a deeper sense of purpose. This enables players to perceive each practice session as intrinsically linked to future competitive success (Le and Wang, 2025). This enhanced sports engagement enabled by technology can serve as a factor influencing satisfaction. Dan et al. contend that sport engagement comprises three components: sport vigour, sport focus, and sport positivity. High levels of sport engagement should manifest in sporting contexts as sunny, contented, and positive emotional experiences, coupled with heightened arousal and a state of positive, focused engagement (Dan and Liu, 2023). Virtual reality technology can fulfil the need for training matches among UBPs, enhancing their sporting experience during exercise. Relevant research has highlighted the impact of players’ sport engagement (Vecina et al., 2012). When players exhibit a high level of sport engagement, they tend to experience greater satisfaction with their participation and a strong desire to continue engaging in it (Valla et al., 2019). Therefore, players’ sport engagement may play a meaningful role in the relationship between VR technology use and satisfaction with training matches for UBPs.

### 5.4 Chain mediation effect of self-efficacy and Sport engagement

The mediation effect analysis conducted in this paper reveals that self-efficacy and sport engagement exert a chain-like mediating effect between VR technology use and satisfaction with training matches for UBPs. The application of VR technology can overcome these constraints by simulating authentic match environments and intricate movement details, enabling students to intuitively comprehend and experience the technical elements of basketball (Narajeenron et al., 2025). It could provide players with controllable success experiences and alternative learning opportunities, providing their self-efficacy. Research within organisational behaviour indicates that self-efficacy positively predicts job satisfaction; the higher an employee’s self-efficacy, the greater their satisfaction with their work (Ismayilova and Klassen, 2019). Furthermore, from the perspective of players’ training and competition, they frequently encounter multiple pressures during both activities, including demanding skill requirements, high performance expectations, and intense competition. These factors may pose challenges to their psychological and physical wellbeing. Players possessing high self-efficacy demonstrate confidence in their performance during training and competition, making them more likely to overcome difficulties and achieve their goals. The sense of fulfilment derived from this confidence can stimulate players’ intrinsic motivation, that may promote their training outcomes and competitive results (Zhang, 2022). Players with high self-efficacy are more inclined to perceive training challenges as surmountable objectives rather than threats, thereby stimulating more intense and sustained sport engagement. This engagement manifests as heightened concentration during training, more positive emotional experiences, and greater conscious effort and persistence (Narajeenron et al., 2025). Consequently, players could undertake higher-quality, more targeted technical and tactical drills alongside psychological adaptation within virtual scenarios (Lafortune et al., 2020). Building upon this, the present study will delve into the relationship between VR technology use and satisfaction with training matches for UBPs, examining the roles of self-efficacy and sport engagement within this dynamic. It will further analyse the underlying mechanisms linking these variables. This holds significant implications for enhancing match performance and satisfaction with training matches. Ultimately, it is through this technology-enabled enhancement of efficacy, which then may translate into a state of deep engagement in training, that players could gain richer skill mastery, situational coping abilities, and positive emotional feedback in simulated matches. These factors may elevate their overall satisfaction with training matches. Therefore, the chained mediation model presented in this study indicates that the relationship between the use of VR technology and satisfaction with training matches is not a simple direct association. Instead, it is primarily mediated through a chained pathway involving self-efficacy and sports engagement. This provides theoretical foundations for the scientific application of VR technology in university basketball training, finding the importance of players’ psychological development and behavioural motivation.

## 6 Limitations and prospects

This study, whilst preliminarily revealing the underlying mechanism whereby VR technology use influences satisfaction with training matches for UBPs through a chain of mediating pathways involving self-efficacy and sport engagement, nevertheless exhibits several significant limitations. (1) The research subjects were typically confined to basketball players from specific universities or regions, with a relatively limited and highly homogeneous sample size. This constrains the generalisability of findings to players of differing skill levels, age groups, cultural backgrounds, or sports disciplines. In addition, all participants in this study were drawn from university basketball representative teams in China. This limitation clearly indicates that the study’s value primarily lies in providing preliminary empirical evidence and theoretical insights for understanding the application of VR technology within the training psychology of a specific group (UBPs). Its generalisability to broader contexts requires future research to conduct cross-validation and comparative analyses across different sports, cultures, and competitive levels. (2) Employing a cross-sectional design with retrospective subjective reporting methods, this approach remains susceptible to individual subjectivity, potentially influencing findings. (3) The predominant use of self-report questionnaires may be affected by social desirability bias, recall bias, or common method bias. (4) The study focused on the chained mediation of self-efficacy and sport engagement, which, while theoretically grounded, may have overlooked other potentially significant mediating variables (e.g., training motivation, cognitive load, presence) or moderating variables (e.g., athlete gender, personality traits, prior VR experience, coaching feedback style). The model may not yet fully capture the complex network of factors influencing satisfaction with technology. These limitations suggest that the current findings represent more exploratory preliminary evidence, with their robustness and generalisability requiring further validation.

Future research in this field holds broad prospects for development and offers multidimensional avenues for deepening exploration. This research necessitates concurrent exploration of the interactions between self-efficacy, sport engagement, and other psychological factors. Incorporating longitudinal tracking designs, cross-lagged analyses, or experimental intervention studies would more robustly reveal causal temporal sequences and long-term effects among variables. Research may extensively employ multimodal data fusion methods, integrating subjective questionnaires, objective physiological indicators, and behavioural performance data to achieve comprehensive, objective assessments of players’ psychological, physiological, and behavioural states. At the level of technological application and personalised practice, with the rapid iteration of VR technology, future developments may yield more intelligently adaptive training systems. These could dynamically adjust training difficulty and feedback based on players’ real-time performance and psychological state, thereby achieving genuinely personalised training. Concurrently, research should expand sample diversity and ecological validity, validating and optimising training protocols across different sports disciplines, competitive levels, and age groups. Ultimately, in terms of practical application, these research findings are expected to provide empirical evidence for universities and professional sports teams. This will guide the scientific design and implementation of psychological skills training and tactical drills incorporating virtual reality technology. The aim is not only to enhance training satisfaction and sport engagement but also to optimise long-term training outcomes and competitive performance, thereby advancing the deep integration of scientific and digital approaches in sports training.

## 7 Conclusion

This study systematically examined the relationship between VR technology use and the satisfaction with training matches for UBPs through empirical investigation and model validation. It confirmed the validity of the chained mediating pathway formed by self-efficacy and sport engagement. Key findings are as follows: (1) VR technology use was significantly and positively associated with the satisfaction with training matches for UBPs. (2) VR technology use indirectly influences the satisfaction with training matches for UBPs via self-efficacy. (3) VR technology use indirectly influences the satisfaction with training matches for UBPs via sport engagement. (4) Self-efficacy and sport engagement exert a chained mediating effect between VR technology use and the satisfaction with training matches for UBPs. These four pathways integrate knowledge from information technology, psychology, and sports science, offering an interdisciplinary exploration of the relationship between VR technology use and satisfaction with training matches for UBPs.

## Data Availability

The original contributions presented in the study are included in the article/Supplementary material, further inquiries can be directed to the corresponding author.
