# Supplementary material for: The effect of virtual reality technology use on satisfaction with training matches for university basketball players: a mediated chain effect of self-efficacy and sport engagement
Source: Front Psychol. 2026 Mar 26;17:1787384. doi: 10.3389/fpsyg.2026.1787384 (PMC13062247; doi:10.3389/fpsyg.2026.1787384)
Supplement: Supplementary file 1 [file Table_1.docx]

Supplementary Material

# Supplementary Table

**Supplementary Table 1.** Fitting indices for the scales.

|  | **χ²/df** | **CFI** | **TLI** | **RMSEA** |
| --- | --- | --- | --- | --- |
| VR technology use | 3.2 | 0.91 | 0.89 | 0.07 |
| Satisfaction with training matches scales | 2.1 | 0.96 | 0.94 | 0.05 |
| Self-efficacy scales | 3.8 | 0.93 | 0.91 | 0.08 |
| Sport engagement scales | 3.5 | 0.92 | 0.90 | 0.07 |
